# Synthesis and Characterization of Folate-Targeted Dextran/Retinoic Acid Micelles for Doxorubicin Delivery in Acute Leukemia

**DOI:** 10.1155/2014/525684

**Published:** 2014-03-02

**Authors:** J. Varshosaz, F. Hassanzadeh, H. Sadeghi Aliabadi, M. Nayebsadrian, M. Banitalebi, M. Rostami

**Affiliations:** ^1^Department of Pharmaceutics, School of Pharmacy and Novel Drug Delivery Systems Research Centre, Isfahan University of Medical Sciences, P.O. Box 81745-359, Isfahan 81746-73461, Iran; ^2^Department of Medicinal Chemistry, School of Pharmacy, Isfahan University of Medical Sciences, P.O. Box 81745-359, Isfahan 81746-73461, Iran; ^3^Department of Biotechnology, School of Pharmacy, Isfahan University of Medical Sciences, P.O. Box 81745-359, Isfahan 81746-73461, Iran

## Abstract

Folate and retinoic acid grafted/dextran (FA-RA/DEX) copolymers with different molecular weight of DEX were synthesized using carbonyldiimidazole and dimethylaminopyridine for targeted delivery of doxorubicin (DOX) in acute myelogenous leukemia (AML). The copolymers structure was confirmed by ^1^H NMR and FTIR. Critical micelle concentration (CMC) of each copolymer was determined using pyrene as a fluorescent probe. DOX was loaded in micelles by the direct dissolution method. Physical properties of micelles, including particle size, zeta potential, drug loading efficiency, and drug release profiles, were examined. The orientation of the folate ligand on the surface of the micelles was studied by X-ray photoelectron spectroscopy (XPS) technique. The cytotoxicity of micelles loaded with DOX at different concentrations was studied in KG1 cells using MTT assay and their cellular uptake by flow cytometry technique. FTIR and ^1^H NMR spectra confirmed successful production of the targeted micelles and XPS spectra showed the surface orientation of folate. R_15_D_10_F_7_ copolymer produced micelles with particle size of 82.86 nm, polydispersity index of 0.3, zeta potential of −4.68 mV, drug loading efficiency of 96%, and release efficiency of 63%. DOX loaded in folate-targeted micelles of RA/DEX was more toxic than that in nontargeted micelles and free drug and seems promising in reducing drug resistance in AML.

## 1. Introduction

Acute myelogenous leukemia (AML) is a clonal disorder of leukemic cells which is characterized by accumulation of blast cells with poor differentiation and proliferative capacity. Standard anthracycline based chemotherapy, such as doxorubicin (DOX), results in approximately 70% complete remission rate in AML patients. Despite the success of DOX against AML, it is associated with significant side effects such as myelosuppression and cardiotoxicity, which can cause irreversible cardiomyopathy and subsequent congestive heart failure [1]. These side effects limit the dose and result in relapse and often development of drug resistance [2].

To reduce some side effects of many anticancer agents like DOX, several nanocarrier systems such as solid nanoparticles, liposomes, liposome nanogel, dendrimers, polymeric micelles, water soluble polymers, and protein aggregates have been developed [3].

Polymeric micelles are composed of a core-shell structure; the hydrophobic inner core encapsulates drug molecules and the hydrophilic outer shell is exposed to the aqueous environment and prevents polymeric micelles from being recognized by the reticuloendothelial system (RES) *in vivo* [4].

Polymeric micelles possess significant properties like biodegradability, small particle size (nanometric range), high loading capacity, extended circulation time, and accumulation in the therapeutic site(s) in the body. Amphiphilic diblock copolymers are the primary type of copolymers used in production of polymeric micelles. These copolymers can organize self-assembly of micelles in aqueous phase [4].

Polymeric micelles have been studied in many clinical trials as carriers for anticancer drugs; for instance, DOX was loaded successfully in micelles of polyethylene glycol/polycaprolactone/Pluronic P105. DOX loading in the composite micelles significantly increased its cellular uptake, improved drug retention, and enhanced its antitumor effect relative to free DOX, thereby providing a novel approach for treatment of cancer [5].

The hydrophilic outer parts should be water soluble, nontoxic and increase the bioavailability of loaded drug in micelles [6]. For this purpose DEX carbohydrate was selected in the present study. It is used mainly as plasma volume expander for more than 50 years. Recently, DEX has been appraised as a macromolecular carrier for delivery of drugs and proteins to enhance circulation time, increase the *in vitro* stability, and decrease the *in vivo* immunogenicity of proteins or enzymes. These have been also used as targeted carriers for drug delivery to specific sites of action via passive or active targeting. Different types of DEX have high water solubility and are available in various molecular weights (Mw). Furthermore, DEX has low cost and contains a lot of hydroxyl groups available for pairing with other molecules. The Mw and charge of DEX affect the distribution and elimination of this polymer. The structure of DEX consists of linear *α*-1,6-glucosidic linkage with some degree of branching via 1,3-linkage [7].

Polymeric micelles can be attached to many ligands such as antibody fragments, epidermal growth factors, *α*_2_-glycoprotein, transferrin, and folate to achieve targeted delivery of drug loaded micelles to cancer cells [4].

Folic acid (Folate) is a vitamin which is essential for the biosynthesis of nucleotide bases and is very consumed by proliferating cells. It has high binding affinity (*k*_*d*_ = 10^−10^ M), low immunogenicity and low molecular weight (Mw = 441.4). It is stable during storage, compatible with many solvents and low cost. Because of these properties it has become a widely used molecule for targeting cancer cells [2].

Three folate receptor (FR) isoforms (FR-*α*, FR-*β*, and FR-*γ*) in human tissues and tumors have been identified. FR-*γ* can be only found in hematopoietic cells [8]. FR-*α* and FR-*β* have been considered as cellular markers for targeted drug delivery because of their overexpression in solid tumors and in leukemia. FR-*β* is extensively expressed in acute AML blast cells. In normal tissues FR-*β* expression is limited to placenta and hematopoietic cells, expressed in the myelomonocytic lineage, and is enhanced during neutrophil maturation or monocyte/macrophage activation. In contrast to AML cells, FR-*β* in neutrophils cannot bind to folate because of aberrant posttranslational modifications.

All-trans-retinoic acid (RA) upregulates FR-*β* in AML cells like KG1 cells [9]. It has poor water solubility and is the most biological active intermediate in the physiologic and metabolic pathway of retinol (vitamin A). This factor plays an important role in the regulation of cell differentiation and stops cell proliferation; thus, it is used in the treatment of various cancers like AML [10]. Considering this advantage of the RA, in the present study a novel colloidal carrier was designed for DOX in the treatment of AML. In this attempt a copolymeric micellar delivery system targeted to folate receptors was synthesized by linking FA to DEX. Then RA ligand was conjugated to the synthesized copolymer backbone in different ratios and the produced micelles were optimized for their physicochemical properties. To our knowledge, there is no report on the production of such copolymeric micelles for carrying DOX in AML. The cellular uptake of the designed novel carrier was compared with nontargeted carrier or the free drug using KG1 cell line which overexpressed folate receptors [11].

## 2. Materials and Method

### 2.1. Materials

Dextran Mw of 6000 and 10000, carbonyldiimidazole (CDI), pyrene, and dialysis tubing cutoff 12 kDa were purchased from Sigma (USA). 5-Dimethylaminopyridine (DMAP), anhydrous dimethyl sulfoxide (DMSO), 3-[4,5-dimethylthiazol-2-yl]-2, 5-diphenyl tetrazolium bromide (MTT), and EtOH were from Merck Chemical Company (Germany). Retinoic acid was purchased from the Solmag Chemical Company (Italy), doxorubicin HCl was from Hangzhou ICH Biopharm Co., Ltd. (Zhejiang, China), and Dulbecco's modified Eagle's medium and FBS were supplied by Gibco Laboratories (USA). The KG1 cell line was supplied by the Pasteur Institute (Iran).

### 2.2. Synthesis of FA/DEX Copolymer

Carbonyldiimidazole (CDI) was dissolved in 5 mL of anhydrous dimethyl sulfoxide (DMSO) followed by FA to produce imidazolide of folic acid (the molar ratio of CDI to FA was 1 : 1). The reaction mixture was stirred overnight at room temperature in the darkness. DEX and 5-dimethylaminopyridine (DMAP) with the molar ratio of 1 : 1.1 were dissolved in 10 mL of DMSO and then the acylation of DEX was carried out by adding 5 mL of the obtained solution of imidazolide to 10 mL of the DMSO solution of DEX and DMAP. The reaction mixture was stirred for approximately 24 hours at 80°C under N_2_ atmosphere in the darkness. FA/DEX copolymer was collected by precipitation in EtOH and filtration. For each 10 mL of the reaction mixture, 50 mL of EtOH was used. Then the precipitate was washed by EtOH and dried at 50°C under vacuum.

### 2.3. Synthesis of FA and RA Grafted DEX Copolymer

To conjugate retinoic acid with FA/DEX block copolymer, imidazolide of retinoic acid (RA) was produced in a similar reaction to imidazolide of FA as described above. The obtained FA/DEX copolymer and DMAP were dissolved in 10 mL of DMSO and then 5 mL of the solution of imidazolide of RA was added to this solution. The molar ratio of imidazolide of RA to DMAP was 1 : 1.1. The condition of reaction and collection of folate and RA grafted DEX copolymer was the same as above. The final product was dialyzed using a dialysis membrane with cutoff 12000 Da against water for 48 h. Then the product was freeze-dried for further 48 h and used for FTIR and NMR tests.

Two grades of DEX with average molecular weight of 10000 and 6000 were employed. The molar ratio of applied DEX 10000, RA, and FA were 1 : 10 : 7, 1 : 15 : 7, and 1 : 20 : 7 and the molar ratio of the DEX 6000, RA, and FA were 1 : 6 : 4.2, 1 : 9 : 4.2, and 1 : 12 : 4.2.  Table 1 shows the exact amounts of the ingredients used in each copolymer.

The structures of produced copolymers were characterized by FTIR and ^1^H NMR spectroscopy of their solution in DMSO-d6. ^1^H NMR spectrum was used to ascertain the degree of substitution (DS). For this purpose, the peaks at 8.7, 3.2, and 1.7 ppm in FA and RA grafted DEX copolymer were used to calculate the ratio of FA, DEX, and RA, respectively.

### 2.4. Surface Chemistry Analysis

The existence of folate ligand on the micelles surface was confirmed by X-ray photoelectron spectroscopy (XPS, X-Ray 8025, Bestec, Germany). The elements on the micelles surface were identified according to the specific binding energy (eV), which was recorded from 0 to 1200 eV with pass energy of 80 eV under the fixed transmission mode. The nitrogen element was particularly tested under fine mode with 0.5 eV as a step. The data were processed by specific XPS software.

### 2.5. Critical Micellar Concentration (CMC) of Folate-Targeted RA/DEX Copolymeric Micelles

The CMC of the micelles was determined by measuring the fluorescence changes of pyrene in different environments with different hydrophilicity. As a hydrophobic fluorescent probe pyrene preferentially partitions to the hydrophobic core of the micelles. When the probe passes from a hydrophilic environment (water) to a hydrophobic medium (micelles core), the excitation spectrum shifts to longer wavelengths. CMC values of different copolymers were determined by monitoring the changes in the proportion of the pyrene excitation spectrum intensities at *λ*_exc_ = 335 nm (*I*_335_) for pyrene in water and *λ*_exc_ = 337 nm (*I*_337_) for pyrene within the micelles core. Excitation spectra were monitored at *λ*_em_ = 390 nm. The micellar aqueous solutions of increasing copolymer concentrations from 2.5 to 100 *μ*g/mL were prepared. And 1 mL of a pyrene solution in acetone (6.0 × 10^−6 ^M) was mixed with 10 mL of copolymer solutions. The final pyrene concentration in each copolymer sample solution was 6.0 × 10^−7 ^M. The samples were shaken 24 hours at 37°C before the fluorescence measurements. Measurements of fluorescence intensity were carried out at *λ*_em_ = 390 nm and *λ*_ex_ = 335 and 337 nm by spectrofluorometer (LS-3, Perkin Elmer, USA). The alterations in the ratio of fluorescence intensity (*I*_337_/*I*_335_) of pyrene with the log (concentration) of FA and RA conjugated DEX copolymers were recorded for finding the CMC [6].

### 2.6. Preparation of DOX Loaded Folate-Targeted RA/DEX Micelles

DOX loaded folate-targeted RA/DEX micelles were prepared by the direct dissolution method. For this method DOX and the copolymer were directly dissolved in distilled water. Loading of the drug into the micelles was done by stirring, heating, and then sonicating the mixture [12]. As reported in our earlier article [13] briefly, the optimized situation for drug loading in the micelles was obtained with 20.1 mg of the copolymer synthesized by DEX with Mw of 10000 Da and CMC < 17 *μ*g/mL, stirring time of 1 h, stirring rate of 560 RPM, temperature of 40.74°C, drug concentration of 15.91%, and then 2 minutes of probe sonication (Bandelin, HD 3200, Germany) using TT13 probe with 30% power amplitude.

### 2.7. Particle Size and Zeta Potential Measurements

The particle size and zeta potential of the folate-targeted RA/DEX micelles were measured by a Zetasizer (Zetasizer-ZEN 3600 Malvern Instrument Ltd., Worcestershire, UK). All particle size measurements were performed in deionized water without dilution using a He-Ne laser beam at 658 nm with a scattering angle of 130°.

### 2.8. Determination of Drug Loading in the Micelles

Drug loading efficiency (LE%) was determined by measuring the concentration of unencapsulated free drug in aqueous medium. For this purpose 400 *μ*L of the drug loaded micelles was centrifuged at 10000 RPM for 15 min in Eppendorf tubes (cutoff 10000 Da) and the concentration of free drug in the aqueous medium was quantified by a UV-Vis spectrophotometer (UV mini 1240 CE Shimadzu, Japan) at *λ*_max⁡_ = 270 nm in distilled water. Unloaded micelles were used as control. Loading efficiency (LE%) was estimated by the following:

(1) $$\begin{array}{c} \text{Drug}\;\text{loading}\;\text{efficiency}\%=\frac{\text{Dru}{\text{g}}_{\text{total}}-\text{Dru}{\text{g}}_{\text{supernatant}}}{\text{Dru}{\text{g}}_{\text{total}}}\times 100. \end{array}$$

### 2.9. **In Vitro ** Release of DOX from Micelles

The *in vitro* release of DOX from micelles was monitored in phosphate buffer solution (PBS) 0.2 M (pH 7.4 and 5.5) containing 2% of Tween to evaluate drug release behavior in response to changes in pH. Six mL of aqueous micellar dispersion of each formulation was placed in the dialysis membrane bags (cutoff 12000 Da, Membra-Cel, Viskase, USA). The open end of the bags were sealed and sunk fully in 15 mL of release medium at room temperature. At appropriate time intervals the concentration of DOX released in the medium was determined by UV spectrophotometry method at *λ*_max⁡_ = 499.4 nm for pH 7.4 and *λ*_max⁡_ = 477.5 nm for pH 5.5.

The parameter of release efficiency within 2 hours (RE_2_%) was used to compare the release profiles [14]:

(2) $$\begin{array}{c} \text{RE}\%=\frac{\int_{0}^{t}y\cdot dt}{y_{100}\cdot t}\times 100. \end{array}$$

### 2.10. Transmission Electron Microscopy (TEM)

Samples of well-dispersed optimum formulation of micelles were placed on a 300 mesh carbon coated copper grid and the grid was left to dry in room temperature. Micrographs were taken with different levels of magnification with an accelerating voltage of 80 kv using a Transmission Electron Microscope (Zeiss, EM10C, Germany).

### 2.11. Cell Culture

Acute myelogenous leukemia (AML) cells of KG1 which show overexpressed folate receptors [11] were used in this study. The cells were cultured on RPMI1640 containing 10% FBS (Fetal Bovine Serum) and 1% antibiotics mixture of penicillin (5000 U/mL) and streptomycin (5000 *μ*g/mL) at 37°C and in 5% CO_2_. KG1 cells are from suspended type cells in culture medium. First 180 *μ*L of the suspension of cells at a density of 5 × 10^4^ cells/mL was seeded into each well of a 96-well culture plate (SPL Lifescience, Korea) and incubated in a CO_2_ incubator (6500, Napco, France) for 24 hours at 37°C in 5% CO_2_ and with proper humidity before the cell viability test.

### 2.12. Cell Viability Assay

After cells were seeded on 96-well plate each row was treated with 20 *μ*L of four concentrations (0.189, 0.377, 0.566, and 0.754 *μ*g/mL) of blank micelles with or without folate ligand, 20 *μ*L of four concentrations (0.189, 0.377, 0.566, and 0.754 *μ*g/mL) of drug loaded micelles with or without folate ligand, 20 *μ*L of the same concentrations of free DOX solution (as positive control), and 20 *μ*L of culture medium (as negative control). Then the plate was incubated for 48 h and after that 20 *μ*L of 5% aqueous solution of MTT was added. After 3 hours of incubation the cell medium was centrifuged (MIKRO200, Hettich, Germany) at 1800 RPM and removed cautiously while not allowing the produced Formazan crystals to pour. Then 150 *μ*L of DMSO was added to the crystals until they were resolved. Immediately after pipetting each row was separately analyzed by ELISA method. Cell viability for each sample was calculated using

(3) Cell  survival% =Mean  of  each  group−mean  of  blankmean  of  negative  control−mean  of  blank×100.

### 2.13. Cellular Uptake

First 720 *μ*L of the suspension of cells at a density of 10^5^ cells/mL was seeded into each well of a 24-well culture plate (SPL Lifescience, Korea) and incubated for 24 hours at 37°C in 5% CO_2_ and proper humidity in a CO_2_ incubator (Galaxy Standard, UK) before the cellular uptake test. After cells were seeded in 24-well plates, each row was treated with 80 *μ*L of each type of blank micelles with or without folate ligand, 80 *μ*L of DOX loaded micelles (0.189, 0.377, 0.566, and 0.754 *μ*g/mL) with or without folate ligand, 80 *μ*L of 0.189, 0.377, 0.566, and 0.754 *μ*g/mL solution of free DOX (as a positive control), and 80 *μ*L of culture medium (as a negative control). Then the plates were incubated for 48 hours and after thoroughly pipetting their contents were centrifuged at 2000 RPM. The supernatant was discarded and 1 mL of phosphate buffer solution (PBS) 0.05 M (pH 7.4) (prepared by adding 50 mL of KH_2_PO_4_ 0.2 M to 39.37 mL of NaOH 0.2 M and adjusting the volume to 200 mL) was added to each Eppendorf tube and centrifuged again. The cells were rinsed twice with PBS and after discarding the supernatant PBS 500 *μ*L of PBS and 2 *μ*L of propidium iodide solution with concentration of 1 mg/mL were added to each tube except one tube as negative control. The tubes were incubated for 15 min in darkness and were studied with flow cytometer (BD FACS Calibur, US) [15]. The cell uptake ratio was worked up from the dot plot graph of forward scattering versus fluorescence intensity. Data were analyzed with the WinMDI program version 2.9.

## 3. Results and Discussion

Considering the cytotoxic effects reported for doxorubicin especially on the acute leukemia and other types of malignancies [16, 17] by directly promoting apoptosis, we tried to see if it is possible to increase its cytotoxic effect by targeting delivery to folate receptors of KG1 cell line using a micellar drug delivery system of FA targeted RA/DEX. As RA upregulates FR-*β* in AML cells like KG1 cells [9], plays an important role in the regulation of cell differentiation, and stops cell proliferation, it was used as the hydrophobic core of the micelles. The copolymers were synthesized by conjugation of FA to DEX followed by grafting of RA to FA/DEX copolymer.

### 3.1. Conjugation of Folic Acid and Dextran

**Figure 1:**
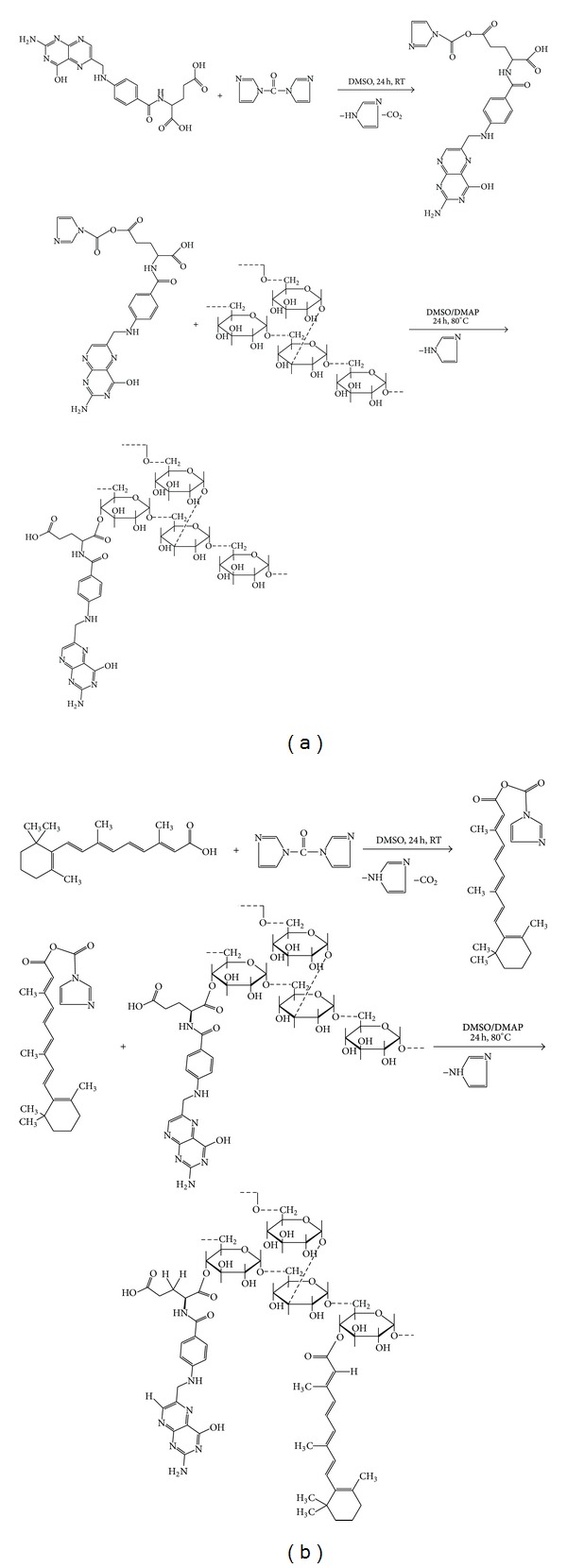
Synthesis of (a) dextran conjugated folic acid (FA/DEX) and (b) FA and RA grafted DEX.

Chemical reaction of FA and CDI, DMAP, DEX, and RA are shown in Figure 1.

**Figure 2:**
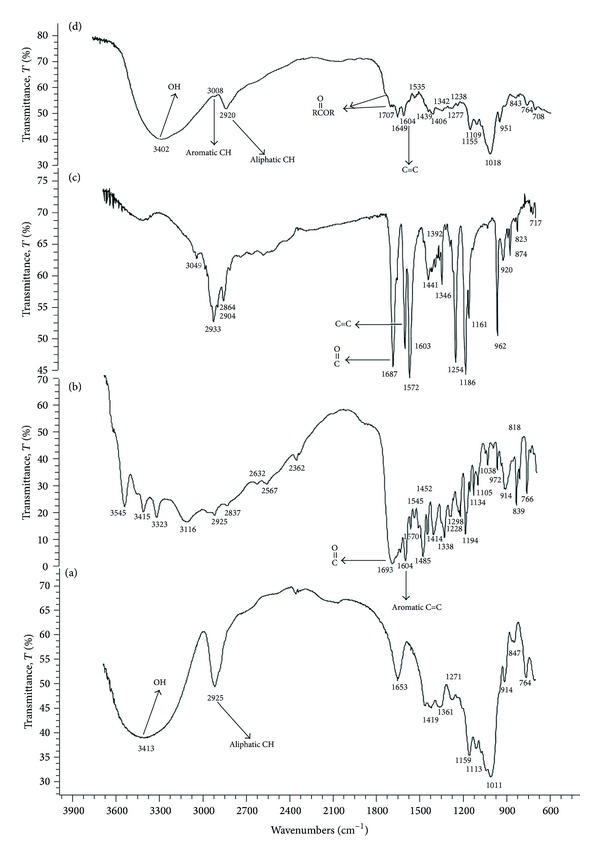
FTIR spectra of (a) dextran, (b) folic acid, (c) retinoic acid, and (d) folate and retinoic acid grafted dextran.

Conjugation of FA and RA grafted DEX was confirmed by FTIR spectra, as seen in Figure 2.

Folate and RA grafted DEX polymer was successfully synthesized by esterification reaction. The emergence of aromatic peak at 3008 cm^−1^ in the FTIR spectrum of FA and RA grafted DEX (Figure 2(d)) in comparison with dextran FTIR spectrum (Figure 2(a)) reveals the presence of RA and FA. The displacement of the peak of the carboxyl group of folic acid (Figure 2(b)) from 1693 cm^−1^ to 1725 cm^−1^ (seen as a small shoulder) indicates the formation of esteric bound between FA and DEX. This small shoulder is due to the small amounts of folic acid. The displacement of the peak of the carboxyl group of RA (Figure 2(c)) from 1687 cm^−1^ to 1707 cm^−1^ indicates the formation of esteric bound between RA and DEX. Actually the lack of any peak or shoulder in 1700 cm^−1^ or higher region in the spectrum of the pure FA (Figure 2(b)) or RA (Figure 2(c)) indicates the change of COOH to esteric form in the product shown at 1707 and 1725 cm^−1^ (Figure 2(d)).

**Figure 3:**
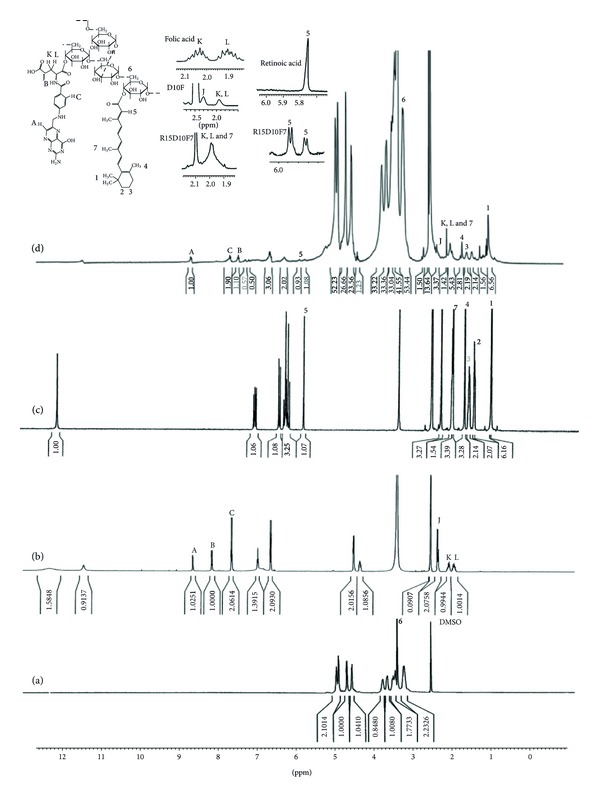
^1^H NMR spectra of (a) dextran, (b) folic acid, (c) retinoic acid, and (d) folate and retinoic acid grafted dextran.

Figure 3 shows the ^1^H NMR spectra of DEX, RA, FA, and their conjugate. There are some new peaks in FA and RA grafted DEX copolymer spectrum (Figure 3(d)) in comparison with DEX spectrum (Figure 3(a)) that are attributable to FA, for example, *δ* = 8.67 (H_A_), 8.12 (H_B_), and 1.99 (H_K,L_). In pure folic acid (Figure 3(b)) H_L_ is observed at *δ* = 1.9 ppm and H_K_ at *δ* = 2.03. In finally grafted compound H_L_ and H_K_ both are observed at *δ* = 1.99 ppm (Figure 3(d)). Because of esteric bound formed between FA and DEX, there is no enantiomeric effect and signals of H_L_ and H_K_ in the ^1^H NMR spectrum are the same. The hydrogen peak of the carboxyl group of FA that is observed at *δ* = 12.351 ppm in pure FA completely disappeared in folate and RA grafted DEX spectrum (Figure 3(d)). Moreover, another reason for conjugation of FA to DEX is the shift of the H_B_ from *δ* = 8.16 ppm to *δ* = 7.45 ppm in the final product (Figure 3(d)).

The ^1^H NMR spectrum of the FA and RA grafted DEX copolymer (Figure 3(d)) has new peaks in comparison with dextran spectrum (Figure 3(a)); for example, *δ* = 7.079 (–CH=CH–C(CH_3_)=CH–C**H**=CH–C(CH_3_)=CHCO–), 6.47 (–CH=C**H**–C(CH_3_)=CHCO–), 5.9 (–C(CH_3_)=C**H**CO–), 2.334 (–C(C$\underline{H_{3}}$)=CHCO–), and 1.022 (–C(C$\underline{H_{3}}$)_2_ of cyclohexenyl) are wholly attributed to RA.

It is obvious that the hydrogen peak of the carboxyl group of RA that is observed at 12.036 ppm completely disappeared in FA and RA grafted DEX copolymer ^1^H NMR spectrum. Hydrogen next to the carbonyl group of RA is observed at *δ* = 5.76 ppm as a singlet. In grafted compound this signal is moved to downfield (5.9 ppm) due to the lower shield effect of esteric bound formed between RA and dextran. In pure RA (Figure 3(c)) signal at *δ* = 5.76 ppm is observed as a singlet as is expected for a hydrogen without neighbor hydrogen, but in FA and RA grafted dextran (Figure 3(d)), this signal is considered as a doublet which could be attributed to the two singlet peaks for each esteric isomer form after conjugation. In addition, the peak of the hydrogen of the nearest methyl to the carbonyl group of RA is also moved to downfield (from *δ* = 2.26 ppm to 2.33 ppm) (Figure 3(d)).

The degree of substitution (DS) of each copolymer is presented in Table 2.

### 3.2. Surface Chemistry Analysis

**Figure 4:**
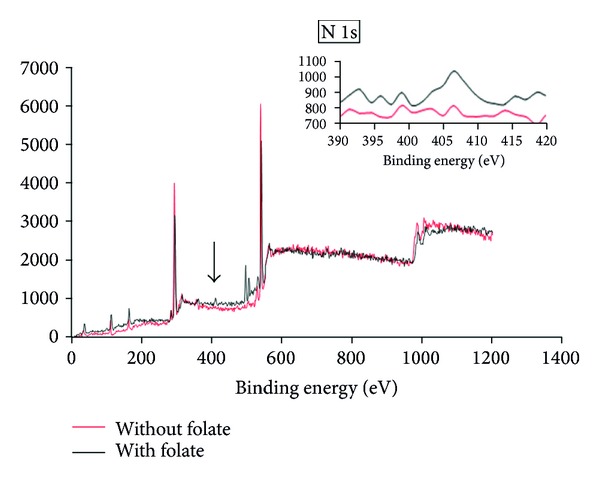
X-ray photoelectron spectroscopy (XPS) spectrum of wide scan spectrum and N 1s peaks from R_15_D_10_F_7_ micelles without and with folate conjugation.

In order to confirm the successful orientation of FA to the surface of the micelles, the surface chemistry of the folate-targeted micelles of RA/DEX was analyzed by XPS to identify the change of nitrogen signal according to the specific binding energy in that one. Pure FA contains 7 nitrogen atoms, while there is no such atom in the structure of nontargeted RA/DEX copolymer. The formulation of R_15_D_10_F_7_ was used for XPS study after preparation of its micelles.  Figure 4 shows the distinct peak of signals from the orbital of nitrogen (N 1s) that presents after the folate molecules embrace the copolymer micelles surface, while nonconjugated micelles present no nitrogen existence due to the lack of amine groups on the surface of the micelles. Therefore, it can be affirmed that the folate molecules have been successfully oriented on the surface of the micelles. The results of Table 2 show the degree of substitution of folate ligand per each mole of DEX. This table shows that there is no quantitative correlation between the surface conjugated folate ligand with the feeding ratio of folic acid, and when 7 moles (in DEX 10000) or 4.2 moles (in DEX 6000) are applied almost a constant amount of this targeting ligand is anchored to the surface of the copolymer which may be due to the steric hindrance and limitations of the available reaction sites. In other words, in the feeding studied ratios of FA and RA it is not feasible to control the surface density of the targeting ligand by changing the feeding ratio in the pairing process.

### 3.3. Micelle Formation and CMC of Folate-Targeted RA/DEX Micelles

**Figure 5:**
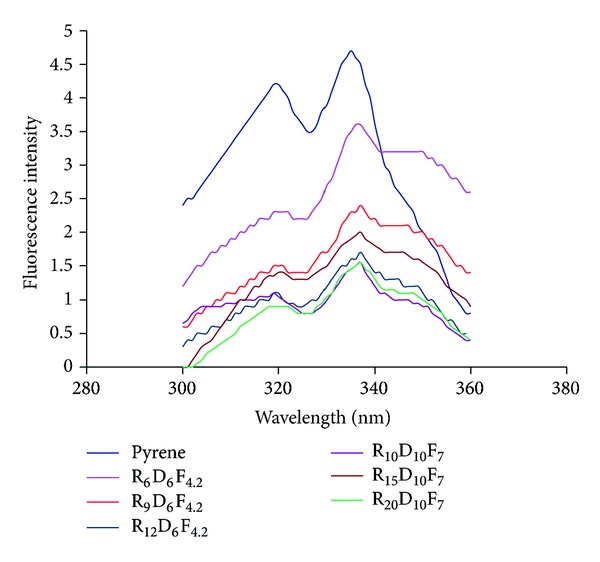
Excitation spectra of pyrene (6.0 × 10^−7 ^M) monitored at *λ*_em_ = 390 nm in the absence and presence of different FA-RA/DEX copolymers.

Pyrene as a hydrophobic fluorescent probe molecule was used to estimate the polymer concentration in which micellization takes place. The excitation spectra of pyrene in water and in the presence of synthesized amphiphilic copolymers are shown in Figure 5.

**Figure 6:**
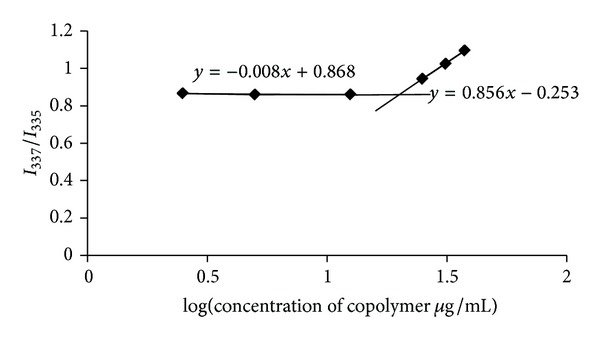
Changes in intensity of *I*_337_/*I*_335_ versus the logarithm of concentration of R_6_D_6_F_4.2_ copolymer.

Plots of the *I*_337_/*I*_335_ ratio versus the logarithm of the concentration of the micellar aqueous solutions are presented in Figure 6. The sigmoidal curves were achieved for these copolymers. For each copolymer solution, the CMC value was obtained from the intersection of the horizontal line with an almost constant value of the ratio of *I*_337_/*I*_335_ and the vertical line with a steady increase in the ratio value. The estimated CMC values are presented in Table 2.

The CMC value of folate conjugated RA/DEX micelles decreased as the ratio of the RA grafted to polymer increased. As the RA grafted to the DEX backbone increased, the hydrophobic moieties of the block copolymer increased, which has a significant effect on decreasing the CMC value. In the aqueous solvents, the hydrophobic RA moieties of the RA/DEX assembled themselves in hydrophobic cores, while these cores were surrounded by hydrophilic DEX backbones, to achieve the lowest Gibbs free energy level. The spontaneous self-aggregation of the RA/DEX in the aqueous medium was determined by the dye solubilization method using pyrene as the probe molecule. The emission spectra of pyrene depend strongly on the polarity of the microenvironment; in polar solvents, the intensity of the first energy band (335 nm, *I*_1_) of the pyrene emission spectrum is higher than that of the third (337 nm, *I*_3_), whereas in a hydrophobic environment, *I*_3_ is higher than *I*_1_. Consequently, when micelles are formed in an aqueous medium, the bran tends to locate itself within the hydrophobic core, increasing *I*_3_ intensity. As a consequence, the ratio of *I*_1_ to *I*_3_ can be used to determine the CMC.

**Figure 7:**
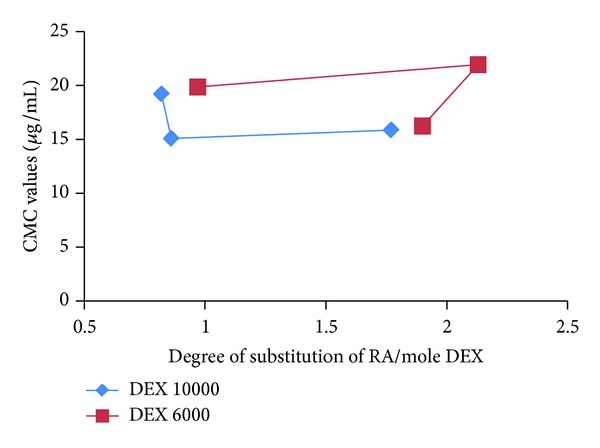
Relationship between CMC values and the degree of substitution (DS) of RA per mole of DEX.

Micelle solutions are diluted in the body fluids; as a consequence, they are expected to be disassociated. Thus, the lower the CMC value is, the more stable the micelles in the body fluids are [18]. Figure 6 shows the variation in the fluorescence intensity ratio (*I*_3_/*I*_1_) against the logarithm of RA/DEX micelles concentration. At concentrations lower than the CMC, the ratios were approximately the same, whereas at concentrations higher than the CMC, a linear increase in the ratios was observed with increasing in the RA/DEX micelles concentration. The CMC values were defined as the crossover point of the two straight lines of each RA/DEX micelle [19]. For DEX molecular weight of 10000, at first the CMC value of RA/DEX micelles decreased as the graft ratio of the polymer increased (Figure 7). Thus, there was a direct correlation between the RA feeding ratio and CMC, at least for the values used in this study. However, with further increase in the degree of substitution the CMC remained constant (Figure 7). This is likely due to the constant capacity of the core of the micelles to accommodate the RA units.

**Figure 8:**
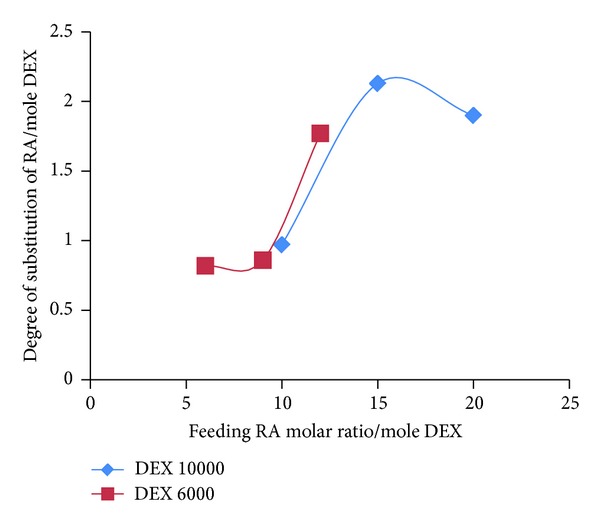
Relationship between the feeding ratio of RA and the degree of substitution (DS) of RA per mole of DEX.

The hydrophobic interaction between the RA moieties of the RA/DEX micelles increased as the graft ratio of the RA/DEX increased, a trend which favors micelles formation at lower RA/DEX concentrations.  Figure 8 depicts the relationship of DS of RA per each mole of DEX and the feeding ratio of RA. As this figure shows, for DEX molecular weight of 10000 Da, as the feeding ratio of RA increases, the graft yield increases and at last it almost gets constant at studied concentrations. This can explain the reduction of CMC values which is then getting constant (Figure 7). For molecular weight of 6000 Da in the studied ratios of RA, at first the CMC was constant, but after that by increasing the RA degree of substitution CMC decreased significantly (Figure 7) which is in accordance with the results of Figure 8.

In this study, the lowest value for CMC of RA/DEX copolymers was about 15 *μ*g·mL^−1^ which is around 155 times lower than the concentrations needed to form micelles with low-molecular-weight surfactants, for example, sodium dodecyl sulphate (SDS) which has a CMC of 2.3 mg·mL^−1^ [18]. This result clearly indicates that the RA/DEX copolymer can form micelles even in highly diluted solutions.

### 3.4. Physicochemical Properties of Micelles

Considering the lower values of CMC for DEX molecular weight of 10000 than 6000 Da (Table 2), this type of DEX with different feeding ratios of RA was selected for further studies. After preparation of DOX loaded micelles by a direct dissolution method their particle size, polydispersity index (PDI), zeta potential, loading efficiency, and release efficiency were studied and the results are presented in Table 3.

Table 3 summarizes the properties of different FA-RA/DEX micelles at 200 *μ*g·mL^−1^ concentration. It was found that the size of these micelles declined in 15 molar ratio of RA and after that increasing the RA feeding ratio was not along with size reduction but an enhancement was observed in the size of the particles. This may be explained in relation to the DS which almost remained constant after 15 molar ratio of feeding RA as seen in Figure 8. The R_15_D_10_F_7_ micelles had the smallest average size of 82.86 ± 1.04 nm. The reduction of micelle size resulted from the enhanced hydrophobic interaction, caused by an increase in the RA grafted to the DEX. In fact, with the increase of RA grafting the hydrophobicity of copolymer was increased and the micelles formed in a more tightly packed core.

The negative surface charge of the micelles (Table 3) is due to the carboxylate groups of FA and RA conjugated groups. While the HCl salt of DOX has made the drug almost water soluble the high loading efficiency of the drug in the micelles is the result of hydrogen bonds between the drug and the hydrophilic corona of the micelles. In all three formulations almost high loading efficiency was observed (Table 3).

**Figure 9:**
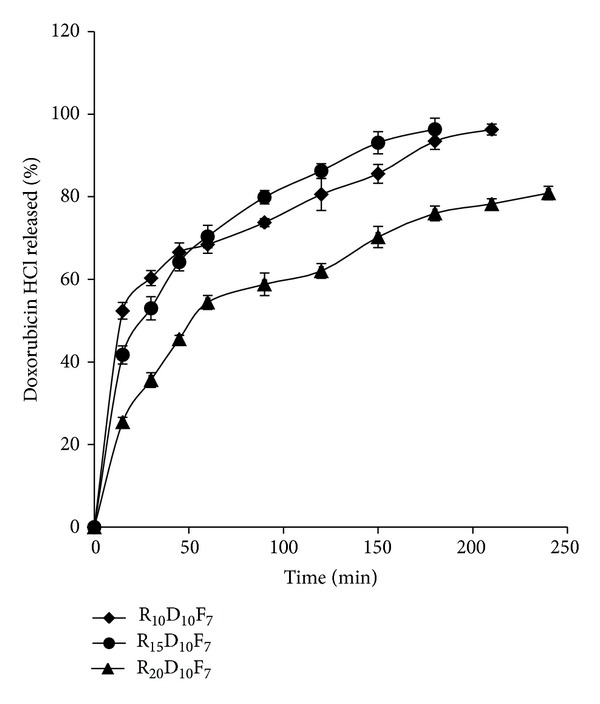
Doxorubicin HCl release profile from different formulations of folate-targeted RA/DEX micelles with different retinoic acid contents at pH 7.4 (mean ± SD; *n* = 3).

Drug release profiles from the micelles are seen in Figure 9. As this figure shows, after a primary burst release of the drug in 20 min, the drug release happened to a near zero-order kinetics and about 90% of the drug was released within 4 hours. The increased feeding ratio of RA caused slower release of DOX compared to other copolymers although the DS has not changed in R_20_D_10_F_7_ micelles.

**Figure 10:**
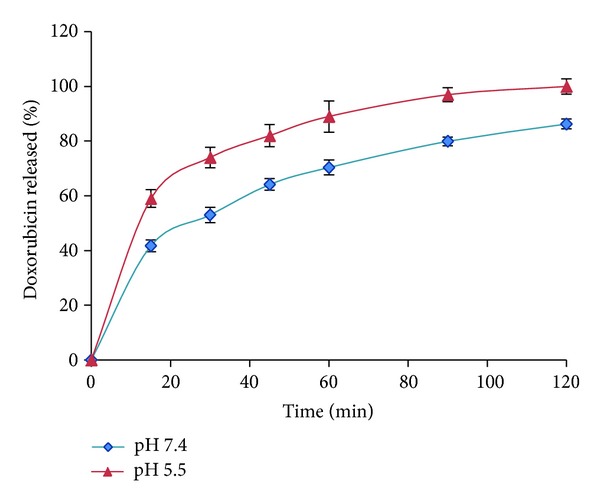
Doxorubicin HCl release profiles from R_15_D_10_F_7_ micelles at different pH.

Figure 10 demonstrates the effect of varying the pH of the release medium on DOX release from the micelles. A reduction in the pH value of the medium increased the rate of DOX release from the micelles. Drug release from micelles was a lot faster at pH 5.5 than at pH 7.4 indicating that after the internalization of micelles into the cytosol of tumor cells the pH of lysosomes may facilitate the release of DOX from micelles.

### 3.5. Transmission Electron Microscopy (TEM) of the Micelles

**Figure 11:**
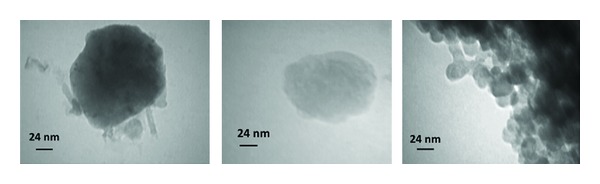
Transmission electron microscopy of the optimized formulation of folate-targeted RA/DEX micelles.

The morphology of the prepared micelles is shown in Figure 11. Although some aggregates of the micelles are obvious in the images, in the field of the micrographs many discrete spherical particles are present. The scale bar of the graphs confirms the particle size of the micelles obtained by DLS method (Table 3).

### 3.6. Cell Viability Assay

**Figure 12:**
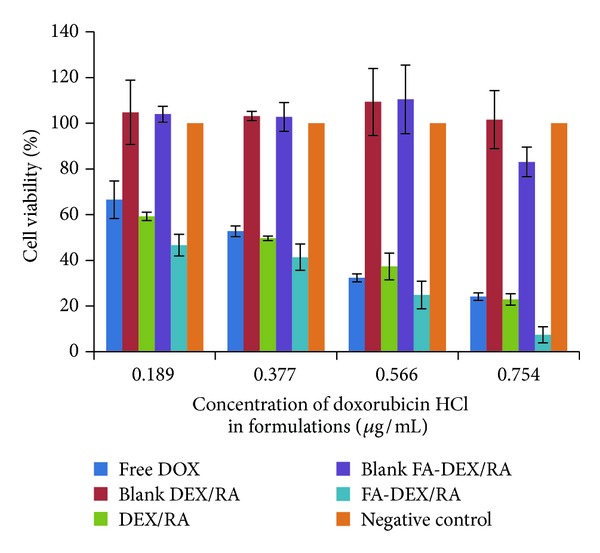
The viability of KG1 cells after treatment with different concentrations of doxorubicin loaded micelles of RA/DEX with or without folic acid conjugate in comparison with free doxorubicin by MTT assay (*n* = 3).

Figure 12 shows the results of cell viability of KG1 cell line by MTT assay. As this figure shows, free DOX in a concentration of 0.754 *μ*M has caused approximately 76% cytotoxicity compared to the same concentration of DOX loaded in targeted micelles that showed about 93% cytotoxicity and nontargeted micelles but with the same concentration of free drug that showed nearly 77% cytotoxicity. The antiproliferative activity of folate-targeted RA/DEX micelles containing DOX HCl was about twice (IC_50_ = 0.325 *μ*M) compared to free DOX HCl (IC_50_ = 0.650 *μ*M). In the concentration of 0.754 *μ*g/mL of DOX there was no difference between the free drug and drug loaded in micelles without folate ligand (*P* > 0.05). Nevertheless, the differences between cytotoxicity of the drug loaded in the folate-targeted micelles and the free drug or drug loaded in the nontargeted micelles were significant (*P* < 0.05). This may be due to upregulation of folate receptors in KG1 cells by RA [9]. Less concentration of the drug showed a similar trend of results but less effective than 0.754 *μ*g/mL concentration.

### 3.7. Cellular Uptake

**Figure 13:**
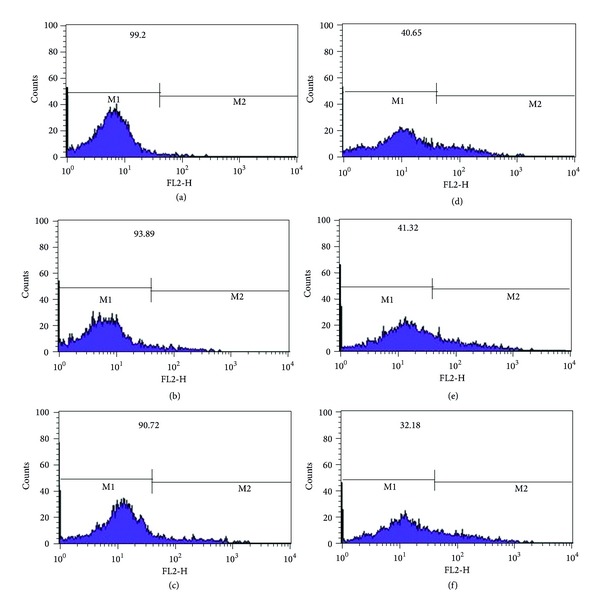
Flow cytometry results to analyze the cellular uptake of (a) negative control (culture medium), (b) blank of nontargeted muscles of RA/DEX, (c) blank of targeted muscles of FA-RA/DEX, (d) free doxorubicin HCl (0.754 *μ*g/mL), (e) nontargeted RA/DEX micelles (containing 0.754 *μ*g/mL doxorubicin HCl), and (f) targeted RA/DEX micelles (containing 0.754 *μ*g/mL doxorubicin HCl). The percentage of viable cells is presented in each image which is computed from the obtained values for the gated cells.

The results of cellular uptake of different treated groups of KG1 cells by flow cytometry method are seen in Figure 13. In a typical flow cytometer, the sample is transported in a stream of liquid through a laser beam and three primary measurements are made: forward light scatter (i.e., cell size), side light scatter (i.e., cell refractivity or granularity), and excited fluorescent dye emission. The fluorescence components can be correlated with the size, construction, and other physical aspects of cells. Suspension cell systems, such as tumor single-cell suspension, are ideal for flow cytometric analysis. To compare the cellular uptake of the drug in different treated groups flow cytometric study was performed. The results of viable cells are depicted on each graph of flow cytometry obtained from the gated percent cells. Figure 13 indicates that for DOX loaded FA targeted RA/DEX treated KG1 cells (Figure 13(f)), fluorescent intensity absorbed by cells increased by roughly 20%, compared to that treated with nontargeted micelles (Figure 13(e)) and free DOX (Figure 13(d)), while there was no significant difference between the free drug (Figure 13(d)) and nontargeted micelles (Figure 13(e)). Figure 13 confirms the results of the MTT assay test.

## 4. Conclusion

FA/DEX copolymer was successfully synthesized by carbonyldiimidazole and dimethylaminopyridine chemistry and RA was grafted to it. R_15_D_10_F_7_ copolymer showed the lowest CMC and was the best formulation. This copolymer produced micelles with particle size of 82.86 nm, low polydispersity index of about 0.3, zeta potential of −4.68 mV, an acceptable drug loading efficiency of 96%, and release efficiency of 63% until 2 hours of release test. The IC_50_ of DOX loaded in folate-targeted micelles of RA/DEX was approximately twice the free drug and seems promising in reducing drug resistance in acute leukemia. We postulate that DOX loaded in DEX/RA micelles that are targeted to folate receptors may serve as a potential strategy to improve the treatment outcome of acute leukemia. This may reduce needed dose of DOX and consequently shortens the required doses which in turn reduces the cardiotoxicity of this drug. The results should be checked *in vivo* to confirm the promising results of the cell culture.

## Figures and Tables

**Table 1:** Dextran Mw, molar ratio, and weight of DEX : RA : FA used in the synthesis of different copolymers.

Copolymer code	DEX Mw	Molar ratio		
		DEX	RA	FA
R_10_D_10_F_7_	10000	0.1 mmol (1 g)	1 mmol (0.3 g)	0.7 mmol (0.31 g)
R_15_D_10_F_7_	10000	0.1 mmol (1 g)	1.5 mmol (0.45 g)	0.7 mmol (0.31 g)
R_20_D_10_F_7_	10000	0.1 mmol (1 g)	2 mmol (0.6 g)	0.7 mmol (0.31 g)
R_6_D_6_F_4.2_	6000	0.1 mmol (0.6 g)	0.6 mmol (0.18 g)	0.42 mmol (0.185 g)
R_9_D_6_F_4.2_	6000	0.1 mmol (0.6 g)	0.9 mmol (0.27 g)	0.42 mmol (0.185 g)
R_12_D_6_F_4.2_	6000	0.1 mmol (0.6 g)	1.2 mmol (0.36 g)	0.42 mmol (0.185 g)

**Table 2:** Degree of substitution (DS) and CMC values of different RA/DEX copolymers.

Copolymer	DS per each mole of DEX		CMC (*μ*g/mL)
	FA	RA	
R_10_D_10_F_7_	3.6	0.97	19.231
R_15_D_10_F_7_	2.42	2.13	15.07
R_20_D_10_F_7_	2.2	1.9	15.85
R_6_D_6_F_4.2_	3.57	0.82	19.86
R_9_D_6_F_4.2_	4	0.86	21.93
R_12_D_6_F_4.2_	3.7	1.77	16.22

**Table 3:** Particle size, zeta potential, loading efficiency (LE%), and release efficiency (RE%) of DOX loaded in folate targeted RA/DEX micelles (mean ± SD, *n* = 3).

Micelle type	Particle size (nm)	PDI index	Zeta potential (mv)	Loading efficiency %	RE_2_%
R_10_D_10_F_7_	98.29 ± 12.66	0.52 ± 0.02	−3.58	93.41 ± 0.97	63.75 ± 1.31
R_15_D_10_F_7_	82.86 ± 1.04	0.35 ± 0.01	−4.68	96.33 ± 3.31	63.8 ± 1.71
R_20_D_10_F_7_	106.6 ± 3.82	0.33 ± 0.05	−7.82	88.9 ± 1.23	46 ± 0.68
